# Tuberculosis “Autoregression”: A Historical Context

**DOI:** 10.4269/ajtmh.22-0590

**Published:** 2022-12-12

**Authors:** Robert S. Wallis

**Affiliations:** Aurum Institute Johannesburg, South Africa Case Western Reserve University Cleveland, Ohio Vanderbilt University Nashville, Tennessee E-mail: rwallis@auruminstitute.org

**Figure 1.**
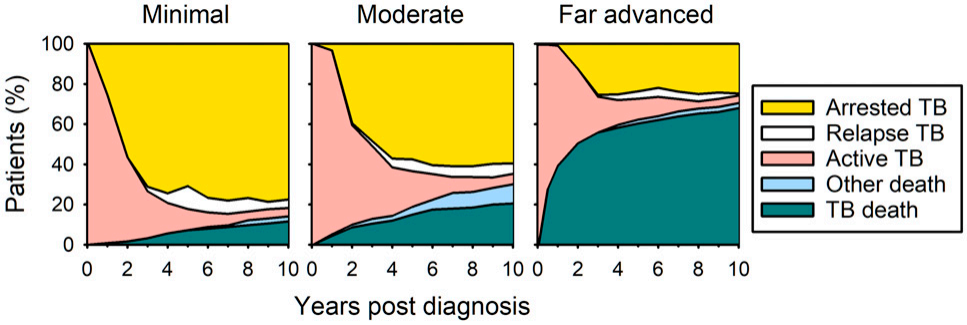
Long-term outcomes of pulmonary TB in the pre-chemotherapy era, according to the radiographic extent of disease at diagnosis.^3^ Relapses occurred when active disease recurred in previously arrested cases. Adapted from reference 2. TB = tuberculosis.

Dear Editor,

I read with interest the case report of Walter et al. describing the apparent spontaneous regression of pleuro-pulmonary tuberculosis (TB) over a period of 18 months in a woman unable to tolerate anti-TB chemotherapy.^1^ I believe several studies from the pre-chemotherapy era can help place this observation in context. The largest relevant case series of which I am aware, published by Alling and Bosworth in 1960, described long-term outcomes in 564 persons who were diagnosed with active pulmonary TB from 1938 to 1948 but did not receive modern chemotherapy.^2^ Outcomes depended on the radiographic extent of disease at diagnosis (Figure 1).^3^ Recovery was most likely in patients presenting with minimal lung disease; this outcome, described as “arrested” TB, required the absence of detectable *Mycobacterium tuberculosis* in sputum, the resolution of constitutional symptoms, the stabilization of radiographic abnormalities, and at least the partial recovery of exercise capacity. However, about 10% of patients who initially appeared to have recovered later relapsed, usually after an interval of 4 or more years. Death due to TB was increasingly likely as the initial radiographic extent of disease worsened.

**Figure 2.**
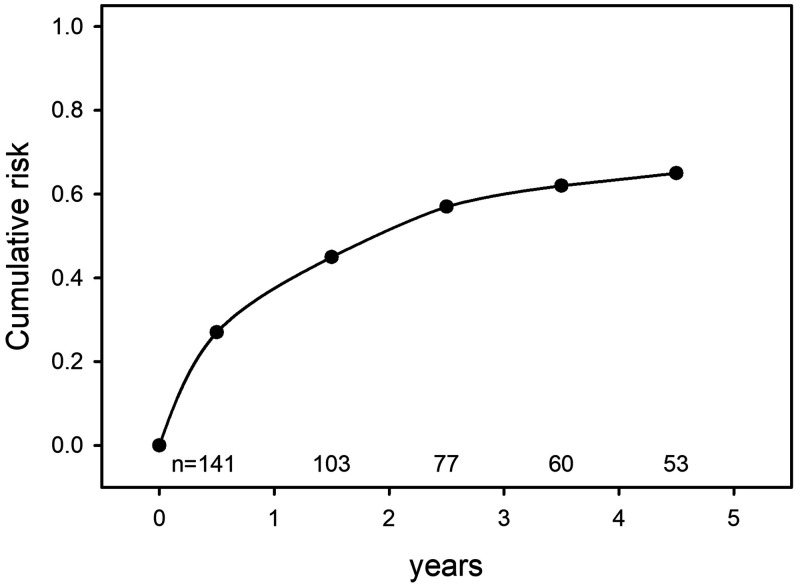
Cumulative risk of tuberculosis (TB) recurrence in US military recruits presenting with serofibrinous effusion prior to the advent of TB chemotherapy. Adapted from reference 4.

A similar literature describes the natural history of pleural TB. In 1955, Roper and Waring reported outcomes in 141 military recruits who had presented 10 years earlier with primary serofibrinous effusion.^4^ The series included only cases thought most likely to be due to *M. tuberculosis* and having at least 5 years of follow-up. Most patients had radiographic and symptomatic improvement following a period of bed rest but were left with some residual evidence of pleural disease. Recurrent TB occurred in 92 patients (65%), over an interval ranging from 9 months to 5 years (Figure 2). Nearly a third of relapse risk occurred after 18 months. Intrathoracic recurrence was most common, although ipsilateral and contralateral localizations were equally likely. Twenty-three instances of extrathoracic relapse occurred, involving nearly every bodily organ, including the eye, larynx, spine and large joints, gut, genitourinary tract, and brain. Some individuals had multiple relapse sites. The wide anatomic distribution of these recurrences is consistent with hematogenous dissemination as the underlying mechanism, occurring despite the apparent resolution of the original infection.

These series may not fully characterize anticipated risks in the patient described by Walter et al., who presented as an older woman with smear-negative pleuro-pulmonary disease in a later century. Nonetheless, they suggest that the risk of anatomically distant TB recurrence remains even years after the apparent resolution of the initial infection, particularly following pleural TB. Better appreciation of the potential seriousness of such recurrences may appropriately influence therapeutic decisions.
